# Civil Service Rules: (Post)Colonial Memoir and the Raj Revival, 1970–1985

**DOI:** 10.1177/03061973241247502

**Published:** 2024-05-21

**Authors:** Sam Goodman

**Affiliations:** 6657 Bournemouth University, UK

**Keywords:** British Empire, Raj Revival, postcolonial, post-imperial

## Abstract

In the 1970s, the India Office Archive within the British Library began inviting the last generation of the Indian Civil Service and Indian Political Service to commit their experiences to written record. Running until the mid-1980s and eventually producing 135 manuscript memoirs, this archive offers a unique insight into the end of the British Empire, as seen a generation hence. This article argues that these memoirs, generated in a time of crisis and fracture within British national identity, are not only vital historical sources but are a significant body of creative work within the context of acute cultural production of narratives of Empire known as the ‘Raj Revival’. Moreover, in their acknowledgement, inclusion and direct dialogue with colonial fictions from Kipling to Forster, as well as their own aesthetic form, these memoirs are part of the long tendency towards the blurring of the boundaries between fiction and life writing within the historical publishing cultures of British India.

## Introduction

One may as well begin with Major Frederick Chauncy's letters to the British Library. As with many of the files of the series Mss Eur F180 and Mss Eur F226, the documents relating to Major Chauncy (Indian Political Service, Chhattisgarh States) contained within are prefaced by a polite letter of reply dated 7 July 1983 and addressed to V. C. Martin (archivist) of the British Library, India Office Library of Records (IOR), then found on Blackfriars Road, London, long before the move to its present site at St. Pancras. Chauncy's reason for writing was in response to a decade-long effort by the British Library to solicit the recollections and experiences of the last generation of Indian Civil Service (ICS) and Indian Political Service (IPS) members, those individuals who had been part of the service from around 1920 or after, and who had not only witnessed and participated in the end of British colonial rule in India in 1947 but who had been observers to the process of Indianisation and the growth of Indian nationalism that preceded it. Chauncy had joined the IPS in 1930 having previously served in the Indian Army (subsequent to representing Britain at the 1928 Olympic Games – he was a hurdler) and remained in India until 1947. As a member of ‘the Political’, he had been part of a small, elite group of civil servants responsible for managing relations between the British government of India, and the Indian rulers of the so-called ‘princely states’ such as those included in Chhattisgarh Division. He was, however, somewhat of an apologetic correspondent, at pains to suggest that, whilst flattered to be invited, he did not believe he had anything worthwhile to contribute as he was always ‘someone's secretary, under-secretary or Personal Assistant’.^1^ As if to underline the point, Chauncy recounts a story of how, at a job interview after Indian Independence, the interviewer looked through his file and pronounced his ‘just a straightforward political career’.^2^

Despite this inauspicious start, and warming eventually to the task at hand, Chauncy goes on to write: ‘Well, if it is of any use, and without going into all the detail suggested by the notes (very kindly supplied), here are the only few items I can think of that might be worth including in any sort of historical record’.^3^ What follows is characteristic of many of the memoirs supplied by Chauncy's co-respondents in the IPS and ICS, offering, as promised, a matter-of-fact summary of the major events of his political career, and his personal perspective on the final years of colonial British India. Reflecting after a couple of pages that he was providing but a ‘poor effort’, Chauncy changes tack, and, abandoning his initial objectivity, goes on to present a variety of anecdotes on those familiar colonial subjects: hunting, drinking and the importance of precedence, as well as the perils of social embarrassment, when dealing with Indian nobility. Though neither the most extensive nor the most illuminating memoir in this repository, Chauncy's recollections illustrate the curious conflict at the heart of colonial life as well as the unique nature of the archive that the British Library sought to create. With a temporal distance of nearly 40 years and a geographical one of 4000 miles, it might have seemed to Chauncy that the events of his Indian life were very far removed from the sleepy market town of Ringwood, on the edge of the New Forest in Hampshire, to where he had retired. However, by the very fact of their creation, and the British Library's drive to gather them, Chauncy's recollections of colonial India indicated how such experiences had been invested with new meaning within the post-imperial context of the 1970s and 1980s, and as the nation sought to contend with its altered political and post-colonial circumstances. Chauncy's account is representative of the character of the memoirs collected by the British Library's efforts, capturing the exhausting extent of the IPS and ICS’ daily work and their governing function alongside the human experience of the insularity of the social life of the British Raj; a close-knit yet intensely hierarchical community, often as disparaging of one another as they were protective.^4^ As such, the papers of Mss Eur F180 and F226 are a vital resource in recording not just the character and ending of the British Empire in India as it was seen by this privileged group of witnesses but also for the insight they give into the change in the wider cultural and historical perception of Empire in the decades after its end.

Eventually running to 135 manuscript memoirs covering the experiences of ICS and IPS officers stationed across India, and who began their colonial service between 1920 and 1940, this archive offers a unique insight into the life of the colonial India in the decades before Indian Independence, and the beginning of the end of the British Empire.^5^ However, the archive, and the ICS generally, is often overlooked within contemporary criticism. Whilst Humphrey Trevelyan, a former District Officer and later British diplomat, sought to reflect on the structure of the ICS (as well as his own family's colonial Indian history) in the early 1970s alongside Terrence Creagh Coen's contemporaneous volume on the IPS, direct critical consideration of the services has been limited in recent decades.^6^ Aside from Clive Dewey's return to the personalities of the ICS in *Anglo-Indian Attitudes: The Mind of the Indian Civil Service* (1993) – a comparative analysis of the contrasting philosophies of District Officers F. L. Brayne and Malcolm Darling and how they reflected the dichotomous British attitudes to India – or David Gilmour's more popular approach to the ICS’ Victorian heyday, the most detailed examination of the history of the ICS, and the wider context of British colonial governance, is found in the work of Elizabeth Buettner and Anthony Kirk Greene.^7^ Greene considers the bureaucratic structure of British rule in three contexts: the ICS, the Sudan Political Service and the Colonial Service, with a particular focus on the District Officer in the field, as opposed to the higher levels of administration. Greene presents an authoritative approach to this history, exploring the personalities as well as the power structures of the colonial governments he considers, however, whilst rich in detail his analysis concludes with the ending of the British colonial presence, and is not a work in which the afterlives and return of Empire within British cultural life is considered. Understandably too, within the frame of such histories of governance, a good deal of the scholarship that is available has focused on the role of the ICS as a blueprint or basis for the structure that succeeded it after independence in 1947.^8^

Buettner, however, chooses to broaden the focus of her work to encompass not only ICS and IPS officers, businessmen, planters and other private citizens of the Raj but, crucially, their families too. Moreover, Buettner's analysis follows these individuals home and considers their circumstances once back in Britain both whilst the Empire was still extant, and after its ending, noting that the drive towards production of memoir and autobiography was in part a quest for ‘proof of existence’ of their Indian experience and a means of substantiating in text the unreal quality of those memories in comparison to their post-colonial lives.^9^ Despite her otherwise nuanced and detailed consideration of the emotional and intimate history of the Raj though, Buettner only notes the creation of the ICS memoirs and the efforts of the India Office Library to collect them in passing, acknowledging their existence within the general drive towards the gathering of such testimony in the 1960s–1980s but not engaging with them directly. Instead, Buettner frames these documents as having value only within a process of nostalgic reaffirmation of Empire, which, whilst not inaccurate in terms of some of their authors’ intentions, nevertheless overlooks their variance, their status within the longer history of the (post)colonial memoir genre, and their contextual significance of their uniquely focused origins. Beyond Buettner, and where historians have explored their contents, these memoirs have most recently appeared as supporting sources in critical and historical examinations of broader conceptual themes relating to colonial experience and the British Empire, mined for their corroborating or anecdotal detail, but again not subject to scrutiny in relation to their creation, composition or literary character as texts.^10^

Responding to this critical field, in this article, I argue that these memoirs are not only vital historical sources that reveal the subjective experience of Imperial service with unusual candour but are a significant body of work within the context of the acute cultural production of narratives of Empire throughout the 1970s and 1980s that was (and continues to be) understood as the ‘Raj Revival’.^11^ Through close textual analysis of these memoirs as well as consideration of their thematic, material and contextual significance, I argue that they are as representative of the return to Imperial mythologies as the fictional texts of the Raj Revival; in their acknowledgement, inclusion and direct dialogue with the colonial fiction of Kipling to Forster, as well as in an awareness of their own aesthetic form, these memoirs are part of the long tendency towards life writing within the historical publishing cultures of British India. Read in light of their literary history though, these texts suggest both continuity and departure, reshaping the purpose of the popular colonial memoir in keeping with the prevailing cultural context as they preserve its traditions; they no longer aim to secure a financial legacy from colonial service for their authors, but instead seek to restore a cultural, and nostalgic, legacy of Empire for the nation and for posterity.

## Forms and Contexts

In order to orient the close examination of this archive, it is necessary to consider its origins both in terms of its material creation and its cultural context. The roots of the British Library's efforts to gather these recollections lie in two connected circumstances; firstly, the acute perspective of their historical moment as British cultural consciousness of Empire began to re-emerge in the 1970s, and secondly, the longer history of Anglo-Indian publishing cultures both fictional and factual, especially the continual prevalence of the colonial memoir over the course of the Empire's existence. As Clive Dewey has argued, in the immediate aftermath of Indian Independence, British interest in the history of South Asia was either non-existent, or, in the context of postcolonialism, focused on the activities of the Indian National Congress or the Muslim League in the years leading up to 1947.^12^ Moreover, Dewey argues, historiographical consideration of the British Raj at this point was considered deeply unfashionable. However, as Britain's fortunes on the global political stage continued to decline and the nation's few remaining territorial possessions gradually secured their independence too, public interest in the vanished world of the Raj and the wider British Empire began to increase once again. Ralph Crane argues that this resurgence is part of a British cultural obsession with India, which has played a major role within the national imagination since the beginning of the British presence there.^13^

Although this fascination may have waned for a period as Dewey suggests, it reasserted itself a generation later with a new-found character and popularity in the form of the ‘Raj Revival’. ‘The Raj Revival’ was the name given to the widespread cultural response to the end of the British Empire observed in the variety of films, novels, television series and other media all fixated with the British colonial presence in India that appeared in significant number throughout the 1970s and 1980s.^14^ In part bolstered by the inauguration of the Booker Prize for literature in 1969 too, this return to the Raj a generation after its ending was a form of cultural production that revived the mythos of British India as much as it recognised its conclusion. It resulted in various productions such as the HBO/Goldcrest adaptation of M. M. Kaye's *The Far Pavilions* (1978), Richard Attenborough's *Gandhi* (1982), the Merchant Ivory production of Ruth Prawer Jhabvala's *Heat and Dust* (1983), David Lean's version of Forster's *A Passage to India* (1984), and ITV's adaptation of Paul Scott's *The Jewel in the Crown* (1984), all of which joined a long list of novels, both literary and popular in nature, that affirm the popularity of colonial texts throughout this period. Such texts spoke directly to those generations of adult Britons who had lived and worked in colonial India or their children, those Britons born in the final years of the Raj; these groups in particular responded enthusiastically to those productions that offered a largely nostalgic view of the colonial India they had known.^15^ However, the Raj Revival's popularity also extended to those audiences who lacked any direct connection with India but who nonetheless felt a kinship with the history of the former Empire in comparison to the diminished state of contemporary Britain.

The significance of these combined political and cultural factors was recognised by the IOR, incorporated recently into the then newly instituted British Library, who began to seek out and encourage former colonial civil servants and administrators to collect their experiences and memories for posterity.^16^ The India Office's intervention, and their efforts to locate, contact and invite these former ICS and IPS men to commit their experiences to paper was a significant, and long-lasting initiative, running for over a decade between the early 1970s and mid-1980s in two parts: the first focused on the ICS, and the latter the IPS. The narratives that the initiative produced were intended primarily for historical record and the benefit of future scholars, and, whilst some authors did write up their memoirs for commercial publication, the majority of the manuscripts in the British Library collections remain unpublished.

The IOR's efforts demonstrate not just a recognition that the experiences of these respondents might, if unrecorded, be lost to history, but also that their recollections and insight into the world of the Raj were possessed of a particular relevance to the time and age and were instructive to the debates over Britain's colonial past, as well as the questions over its post-colonial future. The papers of Mss Eur F180 and F226 exist at the crux of what Ann Laura Stoler identifies as a shift between the ‘archive as source’ into the ‘archive as subject’, with their content and creation inherently linked, each necessary to make the other intelligible and, therefore, of equal significance to this consideration.^17^ Furthermore, their intrinsic connection illustrates how micro and macronarratives of Empire proliferated and informed each other reciprocally in this period, with personal perspectives and experiences informing a general culturally held view of the British Empire that was subject to individual and collective nostalgia. The generation of such archives sought to compensate for an absence where evidence of the otherwise meticulous British habit of record keeping should have been. As Jordana Bailkin and Shohei Sato have separately identified, the impending end of the British Empire sparked the deliberate destruction of documentary evidence in various colonial territories dubbed, fittingly, ‘Operation Legacy’.^18^ Again, such actions validate Stoler's suggestion that the archives themselves should be subject to as much scrutiny as their contents. The replacement of those original documents with a set of commissioned and curated narratives, no matter how candid, must be considered within this larger frame of efforts to sanitise the archival history of Empire, lest the resultant absence grow all the more apparent.

**Figure 1.**
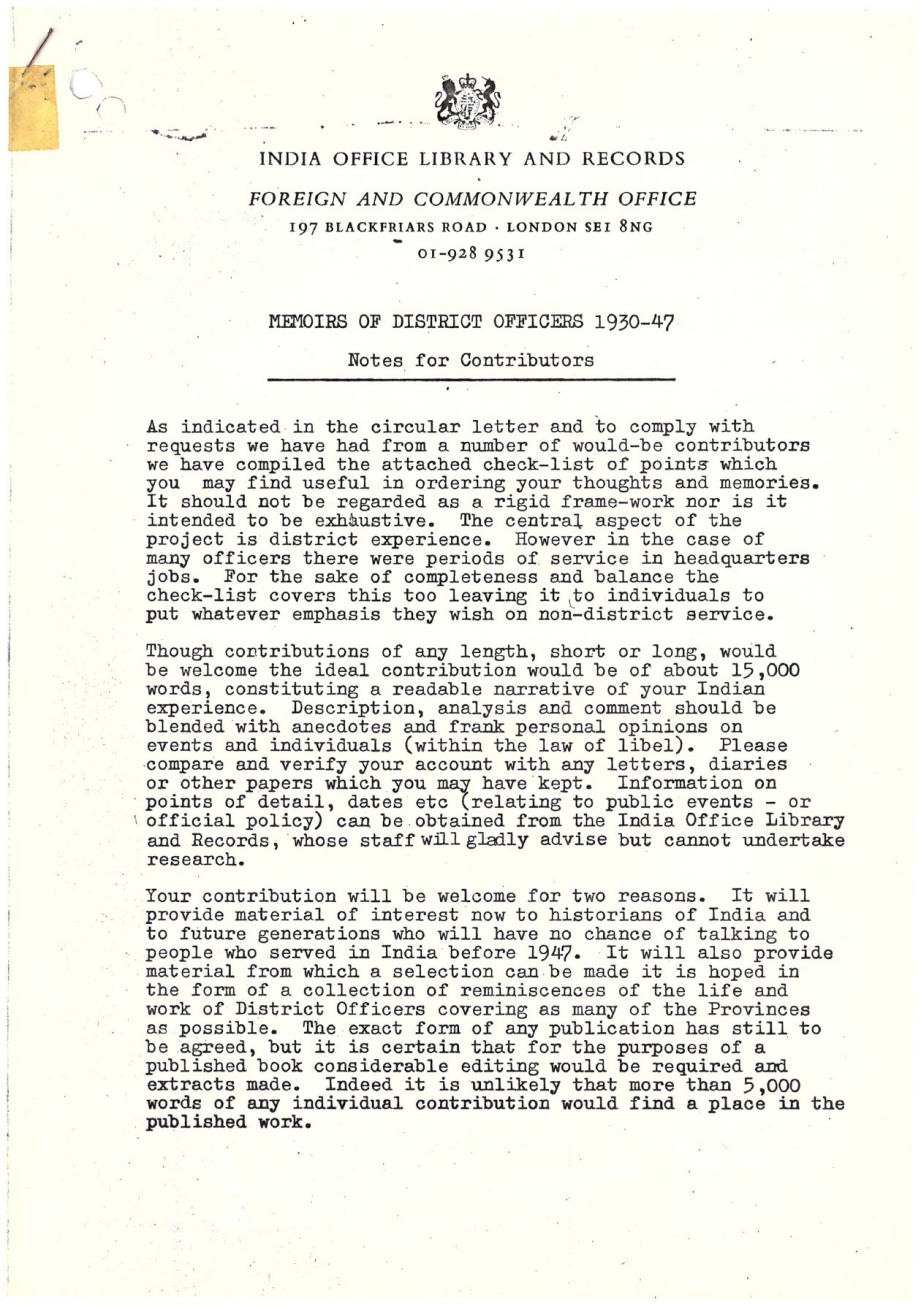
District Officer Notes for Contributors, page 1, British Library, Mss Eur F226.

**Figure 2.**
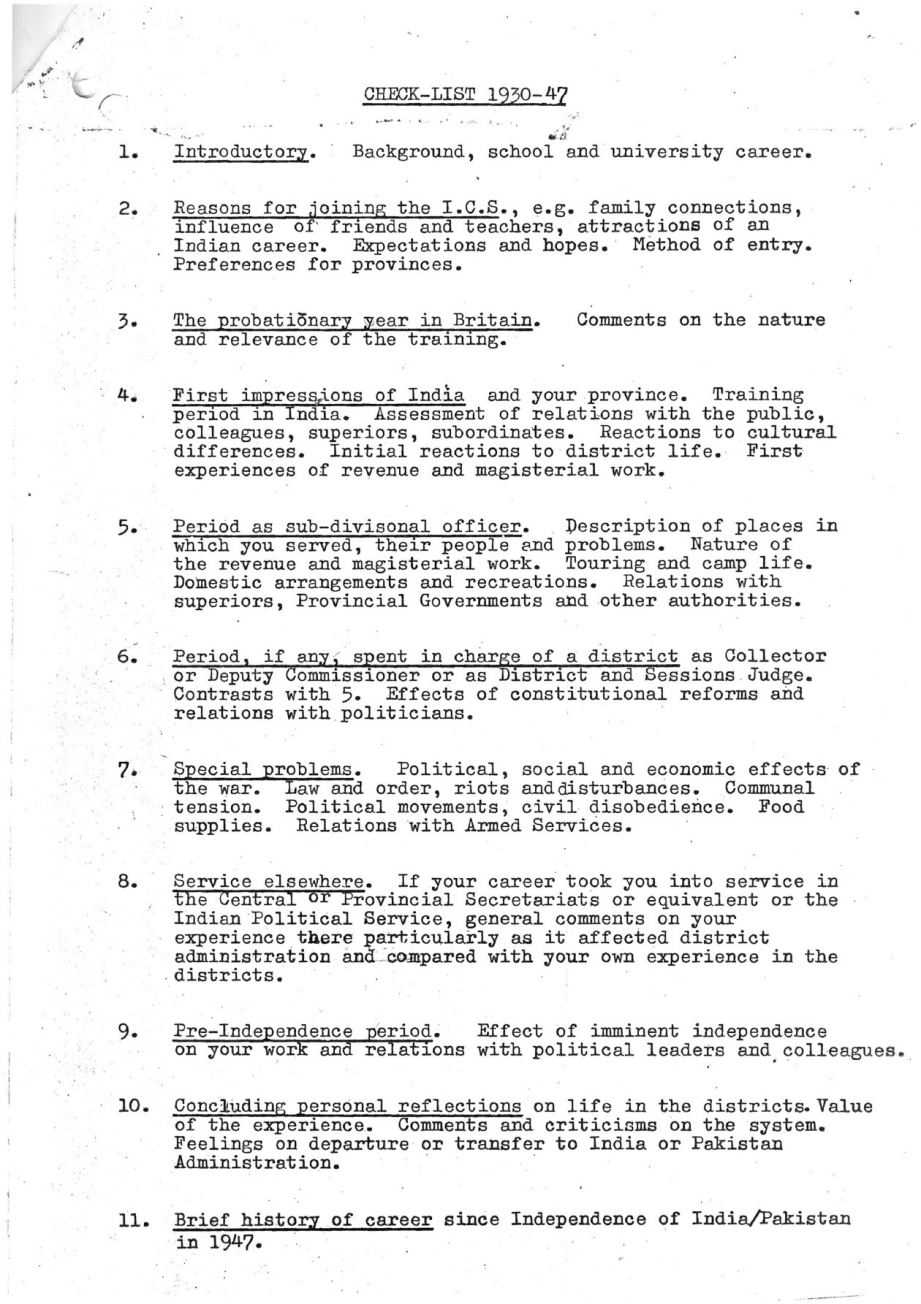
District Officer Notes for Contributors, page 2, British Library, Mss Eur F226.

Whilst the internal decisions and personnel responsible for the creation of the ICS and IPS archive remain unknown, the process through which they were collected and the specifics of how the IOR and British Library directed their contents is well-documented. As noted above, the initiative to generate this archive was really two initiatives, with the approach to ICS officers made via the ICS Association in the early 1970s, and the expansion to IPS officers and their wives from 1981 onwards via the IPS (Retired) Association. Although the British Library no longer has any examples of the original correspondence inviting expressions of interest sent to members, it does retain a copy of the instructions distributed to those respondents who answered these calls within Mss Eur F226 (see Figure 1). This short covering document is significant not only because it adds structural cohesion to the collation of these memoirs within a single repository but also because the terms, language and directions contained within it are generative of a consistency of approach within the memoirs themselves as well as being revealing of the objectives behind the project. As the guidance states, the IOR's emphasis is on producing a ‘readable narrative’ of the respondents’ Indian service, stating that whilst [d]escription, analysis and comment’ are welcome, they should be ‘blended with anecdotes and frank personal opinions on events and individuals (within the law of libel)’.^19^ A justification for this part-scholarly, part-narrative approach is inferred in the following paragraph in that it is the IOR's intention that the collected contributions form not only material that is of interest to historians and future generations who ‘will have no chance of talking to people who served in India before 1947’ but also because extracts taken from a selection of the memoirs would be edited and produced as a published book, presumably for a broad market.^20^ In addition to this general guidance, the Mss Eur F226 file also contains a second page of far more detailed prompts to be used as a ‘check-list’ for ‘ordering your thoughts and memories’ (Figure 2).^21^ Whilst not intended to be prescriptive, most of the respondents follow this structure, many in ordered sections, again creating a consistency to the archive which allows for the consideration of these memoirs as individual texts and a cohesive single textual production for analysis at the same time. Again, these documents illustrate the relationship between individual and collective understandings of Empire, and how the narrative approach to its ending that emerges across fictional and factual formats in this period reinstates it as a subject of textual production and consumption.

In a marked difference to the fictional texts and productions that in-part drove the creation of the IOR archive, it must be remembered that few of the ICS and IPS respondents consider themselves professional authors. Although some developed their memoirs explicitly for publication, and others were lifelong poets or playwrights, the vast majority of them were writing to the brief supplied by the IOR and responded in a manner that it is commensurate with their administrative training. Such guiding contexts are significant, as at first glance, these memoirs appear to conform to the history of amateur publication and authorship that exists throughout the history of colonial India, further aided by the understated process of their creation. However, the appearance of amateurishness conferred by the handwritten manuscripts or the self-deprecating, conversational tone of the covering letters is as misleading as many of the author's claims to lowly functionary status. As with many genres and forms of autobiographical writing, including diaries and memoirs, questions of authenticity, truth and aesthetic quality are often interconnected with audience and the circumstances of a text's production.^22^ In their examination of autobiographical narrative in the late twentieth century, Sidonie Smith and Julia Watson argue that a reader's expectation of the truth available through autobiographical writing requires redefinition, stating that it is a form that ‘cannot be read solely as factual truth or simple fact’.^23^ Instead, Smith and Watson assert a complex relationship between truth and its expression in autobiography refracted through their formulation of the various identities of the writer writing the text. These include the real or historical subject (located in time and place), the narrating subject responsible for writing the text, the narrated subject whose actions and experiences are the focus of that narration, and the ideological subject, namely the prominence of the author's personhood afforded them by the socio-political context in which they write.^24^

Such concerns in turn illustrate the state of autobiographical writing as a cultural and discursive practice; something that existed both as an object of occupation (an aesthetic practice of the writer) and as an object of consumption (by its imagined or actual readers).^25^ Such self-reflexive knowledge in turn informs the composition of the ICS and IPS archive. Aside from apologetic respondents such as Chauncy, some relish the opportunity to give an insight into their experiences, either for their personal satisfaction or pride in having been part of the Government of India, or in the recognition of their place in British history, however, small they might consider it to be. Others meanwhile, as discussed below, follow the British Library's guidance with enthusiasm and dispense a series of anecdotes chained together by occasional mentions of work and a change in posting as structural markers within a wider body of detail.

A recurrent tendency within autobiographies of colonial service is an authorial note that prefaces or begins the main text of the memoir in which the respondent expresses (typically) their amateur status and lack of ability as a writer, as well as a hope that their recollections will be of interest to the reader.^26^ Though evidence of such statements and sentiments exists in this archive and that few of these respondents consider themselves (or have aspirations towards being) professional authors, it must be noted that the ICS and IPS memoirists are a distinctly different group to the typical soldiers, clergy or society figures that might have otherwise written diaries or accounts of their colonial experiences. Preceding amateur memoirists such as Charles Grey, who wrote his own (unpublished and unfinished) memoir of his Victorian soldiering in India late in his life in the 1940s, note the general galvanising effect of the 1870 Education Act on literacy rates on the military and civilian society of Britain and its colonial possessions, resulting in growth of both writers and readers of service memoirs, as well as other forms of popular literature.^27^ The ICS and IPS, however, were recognised as the elite of colonial Indian administration, and whilst few are professional authors, they are invariably highly educated, literate individuals comfortable with communicating complex thoughts, opinions and beliefs in writing as part of their professional routine or personal habits. Indeed, their memoirs illustrate how they were immersed in a world of professional storytelling and narrative creation across their careers.

Directed by point 1 in the guidance notes, many respondents discuss their educational history, particularly their university studies, often noting location (predominantly Oxford and Cambridge, as might be expected), subject studied and their degree classification. Within this overview, 29 of the respondents noted specifically that they studied humanities subjects, mainly History, Classics or Literature, indicative of their facility with language, something further supported by the qualitative nature of the Civil Service Exam, as well as their mandatory year of probation before departure for India, which was spent learning languages, horse riding and other ‘essential’ skills typically at either Oxford, Cambridge or the School of Oriental Studies, London. Before 1914, the entrance examinations for the Civil Service, as David Gilmour describes, had always placed an emphasis on English composition, language, history and literature, with one candidate, Neil Bruniat Bonnarjee (ICS United Provinces) noting how this ‘preponderance for Greats and Classics’, mirrored the public school curriculum, and had always skewed the process against Indian recruits such as his father.^28^ Though reformed and revised during the pause in ICS recruitment during the First World War, and ‘entirely new’ upon its resumption in 1921, the ICS exam nonetheless continued to afford candidates disproportionately high marks based on their facility with language.^29^ The option to choose primarily literary papers remained, with a new format that Cadambi Venkatachar (ICS United Provinces) believed prized the viva voce and ability in verbal reasoning and decision-making.^30^ Confirming Venkatachar's opinion, Victor Matthews (ICS Central Provinces) sat the examination in 1929 and, whilst ‘dismayed’ by the scope of the examination, decided to pick the ‘talky talky’ subjects where he felt there was at least a chance of picking up some marks and little possibility of being absolutely wrong as with sciences, or mathematics.^31^ Matthews goes on to describe how the viva carried a maximum of 300 marks compared to the 100 possible in subject papers, and whilst he made, in his words, a ‘grave strategic error’ in his own viva, nonetheless still garnered 100 points which in combination with his examinations placed him 5^th^ in that year's intake.^32^ Such a process of assessment also supports Dewey's analysis of the ICS in that it was a system that remained sensitive to the stigma attached to ‘cleverness’ by the late-Victorian middle classes, and, whilst seeking academically gifted candidates, continued through the viva to adhere to an ethos where ‘“character” was what counted, not brains’.^33^

Once in post, Dewey states that advancement was conferred on those members of the ICS who possessed the ‘stamina to marshal masses of miscellaneous data, the dexterity to sustain a case through a forest of objections, and a flair for lucid exposition’, again bringing together both personality and ability, and illustrating how this archive indicates a contradiction between the amateur associations of its format and origins, and the professional background of its authors.^34^ Similarly, such herculean feats of administration aside, ICS and IPS officers would keep a tour diary when travelling around the province for which they were responsible. Touring took up a great deal of an officer's time, and involved meeting with village or regional leaders, and hearing grievances or petitions. In larger provinces, touring could take up the majority of the working year; some diarists, such as Nasir Faruqi (ICS Bombay) note that an Assistant Collector was expected to spend 210 days of the year on tour.^35^ Given this itinerant style of living and working, ICS officers would record their engagements, schedule and actions at each stop. The tour diary was thus an official document, and not the qualitative text that might be assumed from its name, but the fact that these men were habituated towards keeping accounts of their movements and daily life is a significant context for the archive of memoirs they would later be invited to produce; it reinforces the idea that the British experience of India always possessed an essentially narrated quality, echoing not only the kinds of travel writing and memoir that were popularised by Empire and colonialism more generally but corresponding to British understandings of India as inherently textually produced.^36^

This state of general linguistic fluency and verbal acuity within the ICS is also reinforced by the working culture and professional hierarchy of the Government of India during British rule, with storytelling and the generation of narrative key to the preservation of that hierarchy. The ICS, itself echoing the East India Company that it succeeded, placed great emphasis on the significance of seniority, the weight of experience and the communication of that acquired knowledge in written or oral form. The result is an occupational and social culture of paternalism and patriarchal power expressed both through these memoirs as textual repositories of experience and within them in the form of the stories and information they contain. The seemingly intimate tone of the memoir, and the personal connections it engenders, are reflective of how the ICS itself was structured and maintained; in a service with limited numbers (never exceeding 1200, across the entirety of India and Afghanistan) and close working often in remote locations, personal relationships were key to supporting the overall smooth running of the service as a whole. There are numerous instances within the archive that indicate how anecdotal experience passed on by senior officers is used to resolve a junior officer's present difficulty or situation, an approach reinforced by the conservative tendency towards tradition and precedent within the ICS as observed by its members such as Trevelyan, and historians such as Dewey.^37^ Such approaches emphasise a culture conditioned towards ‘looking back’, which, in the more general retrospective and nostalgic circumstances of the 1970s and 1980s, affords these texts a further potency and meaning, as well as a degree of historical irony. Caught up in a period of cultural reflection over the circumstances that led to the end of its Empire, Britain, like these amateur authors, turns to its past in an effort to make sense of the present.

## Approaches to Literature

The link between British India and textual production has long been recognised by literary critics and historians, with many noting the ease with which the colonial narrative, and the story of Empire that the British tell themselves, is told and reinforced reciprocally through fiction and factual writing alike.^38^ The ICS and IPS memoirs are thus again of their time, but when read in light of this history of textual production, reflective and responsive to longer narrative traditions of writing on Empire. Led again by the first and second points in the guidance notes, numerous ICS respondents reflect on their connections to India, or their knowledge of it, and how this drove their decision to apply for a career in Imperial service. Along with those born into the Anglo-Indian community, many British respondents cite family connections to India in business, the military or the Civil Service as would be expected, drawing on those connections to shape their own path through their early life, and the initial stages of their governmental career, especially in relation to their choice of province and through maintaining networks of patronage established by relatives and predecessors.^39^ Recognising that the responsibility for the continuation of their family story falls to them, these authors look to write what they acknowledge might be its final chapter whilst they still can.

Others, meanwhile, acknowledge no direct personal or familial link to India, but instead recognise a cultural impulse towards ideals of service and duty inspired by works of popular history, literature and other contemporary publishing cultures. Chief amongst these cultural touchstones are the events and aftermath of the Indian Rebellion of 1857, and the works and influence of the Raj's most successful author, Rudyard Kipling, both of which contributed to the image of an ‘India full of romance’.^40^ Many respondents reflect that the wellspring of their ambition formed from childhood and lingered into adult life. For example, Ian Hay Macdonald (ICS Bihar) applied to the ICS after his graduation from Edinburgh in 1937, writing that he felt genuinely anxious to serve in India whilst such a career was still possible ‘inspired no doubt by the works of Kipling, which as a boy I had read from cover to cover’.^41^ Similarly, Sidney Dunlop (ICS Madras) exemplifies the orientalist expectations inherent to many ICS recruits of the period in his admission that ‘I was influenced by friends of my father; the works of Rudyard Kipling; the prospects of sport; camp life; Indian and Mughal architecture; the Exotic East!’.^42^ Others more openly reflect the prejudicial attitudes of previous generations; Fraser Noble (ICS North-western Frontier) reflected how reading G. A. Henty ‘began to stir in me the interest in backwards countries and their peoples which finally emerged from my subconscious with my service in India’.^43^ In parallel to literary inspirations such as Kipling or Henty, the events of the Indian Rebellion of 1857, or the ‘Mutiny’ as it was long known, exerted an intense influence over the actions of the Government of India, and the Indian Army for the remaining 90 years of the Raj, and featured continually in various popular cultural outputs of Britain and British India alike. Chief amongst these were numerous service memoirs and diaries written by British civilians, soldiers and journalists sent to report on the fighting, but the Rebellion also inspired numerous fictional works, including many within the context of the Raj Revival of the 1970s.^44^ As Eric Midgley (ICS United Provinces) states:

‘When I was very young the Mutiny was still alive in English folklore. My grandfather used to play on the piano a dramatic piece entitled “The Relief of Lucknow”. This was a series of themes introduced by captions. “General Havelock's forces are sighted – tara, tara”. “The Mutineers flee – hurry music”…’.^45^

Midgley's observations on the longevity of the Rebellion within the British psyche are echoed by other respondents, including Samuel Solomon (ICS Bihar) who writes of how he used to mark the ‘Mutiny’ annually on the 10^th^ May, ‘an event that had been fixed in my boyish mind by the image of Miss Holland's ancient mother at Hampton Court School, who had actually lived through the Mutiny’.^46^ The ‘magnetism’, as he puts it, of the Rebellion leads him to request the United Provinces, site of the major sieges of Lucknow and Cawnpore, as his preferred first choice of posting on his acceptance into the ICS.^47^ Solomon and Midgley's accounts suggest the interlinked nature of cultural and social understandings of Empire, as although without a family tradition of Indian service, they nonetheless see themselves as inheritors of such broader national historical narratives, and, in their repetition of them, fulfil a responsibility towards preserving them for future generations.

Adherence to such narratives is not always uniform. As might be anticipated in an archive of this scale, the degree of belief in the principles of Empire as a motivation for joining the ICS varies, from those respondents who sought the idealised image garnered from their reading habits through to those who expected the ICS to provide financial and career security, to those who believed it was their responsibility to continue British rule for as long as it might possibly last. Some ICS officers note an agnosticism borne out of their university education, especially where their undergraduate or probationary years coincided with some of the more contentious Oxford Union debates over national identity and allegiance in the early 1930s such as ‘India is under no obligation to remain within the British Empire’ and ‘This House will not fight for King and Country’, or the passage of the Government of India Act in 1935.^48^ However, despite these notes of dissent, when presented with criticism there is a notably consistent response. The other significant literary reference within these memoirs is to E. M. Forster, specifically the effect and perception of his novel *A Passage to India* (1924).^49^ Joseph Thompson (ICS, Madras) describes how his superior had been ‘incensed’ by Forster's novel and how it had turned District Officers into a ‘caricature’; he goes on to record the views of a subsequent Collector, ‘a brilliant Brahmin who had been Third Wrangler at Cambridge’, who felt it ‘wicked and mischievous. Grossly libellous to both Indian and Englishman’.^50^ Samuel Solomon meanwhile mentions how he meets during his probationary year at Cambridge Lytton Strachey, A. E. Housman, John Maynard Keynes and, years later, E. M. Forster, whom he ‘accosts’ in order to enquire:

‘[W]hat precisely had transpired within the murky depths of the famous Marabar Caves – a climactic incident leading to the conviction of the innocent Aziz. He was put out by my impertinence and suggested I read it again. I did, but whether it was my legalistic training for the I.C.S. that found holes in his narration, or mere obtuseness on my part, I remained unconvinced’.^51^

Just as Solomon undermines the validity of *A Passage to India*'s critique by questioning the logic of Forster's plotting, Thompson similarly dismisses it as a ‘nine-day's wonder’, asserting that a district official in India bore no resemblance to Forster's ‘caricatures’.^52^ Even the one positive response to Forster becomes an implicit piece of criticism. Hugh Lane (ICS, United Provinces) indicates the book's formative influence on him: ‘I had been much influenced by E. M. Forster's ‘A Passage to India’ and I was imbued with the conviction that the British in India were far too aloof, race-conscious, and “superior”: I was anxious to prove I could be friendly with anyone’.^53^ Lane is warned off such an approach by his District Commissioner but proceeds to socialise with Indians he meets via a local tennis club, only to discover to his dismay that a Hindu shopkeeper he had thought was his friend was only inviting Lane to dinner to gain prestige from their association. Such collective resistance to Forster's criticism of Anglo-Indian society is ironic, as it serves to substantiate exactly the attitudes Forster satirised in his characters of Turton and Heaslop, an embodiment of the dismissive and insular aspects of Raj society, and its hostility to those it saw as outsiders.

As might be expected, the Indian ICS memoirists in this archive do not share the same predisposition towards the literatures of colonial India as their British colleagues, and instead see in Kipling's version of the Raj no place for themselves, and so wish to correct it, or use its ideals as a basis for further critical reflection on its practices. Cadambi Sheshachar Venkatachar (ICS, United Provinces) takes issue with the belief, expressed throughout many of the other memoirs here, that British Indian society was progressively integrated, and the ICS effectively a society of equals across ethnic lines. Venkatachar writes ‘[t]he fact was the British and Indian did not ‘connect’. In the twenties, E. M. Forster's Englishman Fielding asked the Muslim Aziz: “why can’t we be friends now”. A hundred voices said, “No, not yet”. The same echoed when the British left in 1947; the Indian sly [sic] repeated: “No, not there”’.^54^ For all the efforts of Thompson or Solomon to tell a nostalgic story of British colonial benevolence, and a continued association of friendship between Britons and Indians, the insights of respondents such as Venkatachar suggest a far less inclusive parallel reading of the same account, and one akin to the process of writing back to be found in the postcolonial literature of the period.

This interplay between literature and culture, as well as the production and transmissibility of narratives of Empire here, is a significant force within these documents that influence not just the motivations these authors but the character, form and limits of their writing. The importance of Kipling, Henty and the ‘Mutiny’ narratives (in addition to the consistency of educational background, in level if not always location) is not simply confined to their recurrence as common reference points that suggest a shared cultural vocabulary but rather that their use and reuse reinforces a discursive regime of power that privileges a prejudicial view of India and Indians both in the narrative present of the memoirs and the post-imperial era in which they were created. The British ICS respondents and their writing demonstrate the resilience and longevity of the Kiplingesque view of India in so much as their exposure to such representations in childhood creates an expectation that they go on to attempt to fulfil through their service; despite finding, as these texts reveal, that the India they expect does not truly exist, many nonetheless fall into its tropes and reinforce the same orientalised description of it via the act of their own writing decades later. The deep-seated nature of such attitudes is exemplified in respondents such as Richard Slater (ICS Punjab) who noted a strange sensation of homecoming on arrival in India in which he writes:

‘Memory could have played no part…I must have absorbed during my twenty years of conscious life far more than I realised of the India-related literature, photographs, pictures and miscellaneous momentos [sic] which were still part of the cultural heritage of so many of my generation, whether or not their links with India were direct’.^55^

Alternately, the Indian respondents in this archive, whilst being unable to identify with this same canon of colonial literature in the same way as their British colleagues, are nonetheless also influenced by it. The dominance of the roles, metaphors and perspectives drawn from the literary culture of the Raj mean that these Indian memoirists are depicted as effective outsiders within the inner-circle of the ICS, often taking on the mantle of the disaffected observer (or narrating subject) as opposed to being truly part of the events and society they describe. Thus, their perspectives, though critical of the tropes of colonial literature and culture, often reify them in the same way as writing a memoir serves to perpetuate the culturally held views of the British Empire itself.

As a consequence of these literary influences upon them, these works sit at an intersection of creative non-fiction and historical record, effecting a body of documentary evidence that enables analysis of the subjective experience of the end of the British Empire, but in a fashion that is alert to literary sensibilities, genres and techniques. The framing context of the British Library's efforts and the memoir format mean that these texts cannot be read as examples of autofiction despite the guiding emphasis on the production of a ‘readable narrative’; unlike contemporaneous authors such as John Masters or Paul Scott whose experiences in the Indian Army were translated into novels overtly, these texts are presented as faithful narratives of witness.^56^ However, given their indebtedness to the tropes of colonial literary production as well as the emotive nature of their subject matter, they cannot be viewed as sitting entirely within the genre of non-fiction and the pretence of objectivity that it might suggest either. In their emulation of the imagery, language and metaphorical lexis of writers like Kipling or in their engagement with Forster's questions of identity, these memoirs reflect many of the tropes of colonial fiction's plotting and narrative modes also, as exemplified by Slater's enactment of wonder in his arrival at the Indian dockside. Instead, recalling Smith and Watson's assertion, these texts are a more complex blend of subjective truth, and historical experience.

The scope of these memoirs too, structured by the India Office Archive's guidance notes, meant that they often cover an entire lifetime's experience, ranging from childhood memories through to the contextual present of the 1970s and 1980s, when their authors are either in or nearing retirement.^57^ This broad temporal span suggests a further confluence between the fictional and factual as these memoirs appropriate the form and content of a Bildungsroman. The Bildungsroman, as Petru Golban argues, is a work whose central theme is that of identity formation but remains a genre characterised by its essential hybridity.^58^ The memoirs of the ICS and IPS conform readily to such a description, bringing together a narrative of how their authors develop from youth to adulthood through their experience of different institutions and political contexts, but also reflect the various formative influences of the cultural texts of Empire and the effects they have on these authors’ style, idiom and consciousness and their perpetuation of the colonial memoir. The significance of these works as narratives of identity within a period of enormous change and redefinition to ideas of Britishness is likewise key. Building on Franco Morretti's consideration of the genre, Sarah Graham argues that the Bildungsroman ‘is profoundly concerned with what it means to be an individual and to participate in the life of a nation’; in the context of the end of Empire from the 1940s onwards as well as its cultural revival in the 1970s, the implication of Graham's analysis is that these texts may be read as part of a personal process of critical reflection, but also a national, post-imperial one, again placing them within the backwards-looking efforts of the ‘Raj Revival’.^59^ Similarly, as well as recording the personal markers of self-determination as anticipated by the memoir format such as their actions and notable achievements, these texts cannot but be influenced by the vicissitudes of imperial and post-imperial history; whilst not, as per Ericka A. Hoagland's formulation, a concerted ‘political act of counter-colonisation’, the postcolonial elements present in the memoirs of Indian ICS authors are evident.^60^ Writing with the benefit of 30 years of reflection, these authors are configured as omniscient narrators in a position to evaluate the beliefs and development of their younger selves and the world that produced them, leading, hopefully, to a place of contemporary enlightenment, and not just nostalgic reminiscence.

## Conclusion: The Sense of an Ending

As this article has demonstrated, the collection of ICS and IPS memoirs held by the British Library's India Office Archive is somewhat of a contradiction. On one hand, they are, to echo the description of Frederick Chauncy's Indian experience, a ‘straightforward’ autobiographical record of a particularly privileged section of colonial society; they present a familiar picture of British India in the 1930s and 1940s, offering eye-witness accounts of its major events and asserting, generally, that the men of the ICS and IPS had the best interests of Indians and Empire at heart in their actions. On the other hand, they are a peculiarly revealing set of sources, singular by the fact of their deliberate creation through the British Library and the consistent internal structure afforded by the guidance notes, as well as because of the depth of personal detail they provide on the government of India, especially where the history of such subjects has traditionally focused on structure, hierarchy and process and not human experience. Rarely too in colonial memoir is such a sustained body of sources from the same social group collected in such a fashion, other than in the instance of exceptional events such as the Indian Rebellion, or either of the world wars. Moreover, as a result of the paratextual circumstances of their production, these memoirs are as much a time capsule of the 1970s–1980s as they are recollections of Empire of the 1930s–1940s, capturing how their authors thought and felt not just in the moment of the actions they describe but also at the end of their careers, looking back over the changeable fortunes of Britain and its former Empire in British cultural and political life over the course of the late-twentieth century.

This connection to the cultural narratives of the 1970s and 1980s means that these texts are produced within, and are indebted to, the ‘Raj Revival’, the name given to the resurgence of interest in the stories, history and nostalgic reminiscence of British India in this period. The framing context of the Raj Revival pervades this archive, meaning that its component texts are not just part of a movement towards historical revisionism but are rather part of a process of cultural rehabilitation of Empire and its history; in part a response to the postcolonial discourses of geopolitics, scholarship and literature that had emerged since the beginning of the end of Empire in 1947, these memoirs bring together individual and collective narratives of the British past, showing the ease with which the two intersected and supported one another and have allowed for the development of a deeply embedded nostalgic narrative of Empire that continues to the present.^61^ Similarly, through their connection to these culturally produced narratives of Empire, these texts appropriate the subjects and modes of expression associated with the literatures of the Raj too. Through their acknowledgement of the influence of writers such as Kipling, the foremost author of the Imperial heyday, or in their efforts at presenting a rebuttal to Forster, one of the Raj's most prominent literary critics, these texts are further evidence of how the distinctions between colonial literature and colonial history, between fictional and non-fictional accounts of Empire, are far thinner and more porous than usually assumed.

Of course, the contents of such an extensive repository cannot be explored in their entirety in a short article such as this, and I have not been able to consider many of the prominent themes of these texts in as much detail as they warrant. There is far more work to be done on their contents and in reconstructing the social, domestic and interior lives of these authors, not to mention the insight they give into the political history of the final decade of the Raj and the encroaching changes brought by the Second World War. However, in light of the various historical and cultural returns that this article acknowledges and to go back to where it began itself, the article has developed the scholarship on the historical record of British India and of colonial memoir more broadly to demonstrate that when it comes to the recollection of the British Empire, nothing is as ‘straightforward’ as it might first appear, or as its memoirists might wish to tell you.

## Notes

1. Frederick Chauncy, Mss Eur F226/6, British Library, 1.

2. Chauncy, F226/6, 1.

3. Chauncy, F226/6, 1.

4. See Dennis Kincaid, *British Social Life in India 1608-1937* (London, 1938).

5. Recruitment to the ICS and IPS was suspended with the beginning of the Second World War and never resumed due to the evident imminence of Indian Independence after 1945. Those recruits who had progressed to their probation year by 1939 were, however, able to begin service in 1940.

6. See Humphrey Trevelyan, *The India We Left: Life as One of the Last Generation of British Officials in India* (London, 1972); Terence Creagh Coen, *The Indian Political Service: A Study in Indirect Rule* (London, 1971).

7. See David Gilmour, *The Ruling Caste: Imperial Lives in the Victorian Raj* (London, 2005); Clive Dewey, *Anglo-Indian Attitudes: The Mind of the Indian Civil Service* (London, 1993); Anthony Kirk-Greene, *Britain's Imperial Administrators* (Basingstoke, 2000).

8. See P. N. Parashar, *Histories and Problems of Civil Services in India* (New Delhi, 2003).

9. See Elizabeth Buettner, *Empire Families: Britons and Late Imperial India* (Oxford, 2005), pp. 256–257.

10. See Andrew Rotter, *Empire of the Senses: Bodily Encounters in Imperial India and the Philippines* (Oxford, 2019).

11. For a history of this term see: Andrew Teverson, *Contemporary World Writers: Salman Rushdie* (Manchester, 2007), p. 7.

12. Dewey, *Anglo-Indian Attitudes*, p. 8.

13. Ralph J. Crane, *Inventing India: A History of India in English Language Fiction* (London, 1992), p. 5.

14. Alongside Teverson's overview, Rushdie's original article provides numerous points of cultural reference on the development of this term. See Salman Rushdie, ‘Outside the Whale’, *Granta*, 1 March 1984. https://granta.com/outside-the-whale/ (accessed 27 March 2024).

15. Although it was the lighter fare that typically attracted popular success and accolades, such as Booker prizes for both J. G. Farrell's *The Siege of Krishnapur* (1973) and Scott's *Staying On* (1977), the Raj Revival was not without those texts that asked more serious questions of the British Empire in India, such as the depiction of racism and abuse of power by Merrick in Scott's *Raj Quartet* (1966–75), or the ostracism of Olivia after her interracial affair and her abortion in Jhabvala's *Heat and Dust* (1975).

16. The IOR's approach may have been influenced by Noble Frankland's work at the Imperial War Museum in the 1960s to record oral histories of experiences of the Second World War, later used in the ITV series *The World At War* (1973), or by the contention raised by the documentary *The British Empire*, first broadcast on the BBC in 1972.

17. Ann Laura Stoler, *Along the Archival Grain: Epistemic Anxieties and Colonial Common Sense* (Princeton, 2009), p. 44.

18. See Jordana Bailkin, ‘Where Did the Empire Go? Archives and Decolonisation in Britain’, *American Historical Review* 120:3 (2015), 884–899; Shohei Sato, ‘Operation Legacy: Britain's Destruction and Concealment of Colonial Records Worldwide’, *Journal of Imperial & Commonwealth History* 45:4 (2017), 697–719.

19. British Library, India Office Records, ‘Memoirs of District Officers 1930-1947: Notes for Contributors’, Mss Eur F226, 1.

20. BL, ‘Memoirs…’, Mss Eur F226, 1.

21. BL, ‘Memoirs…’, Mss Eur F226, 1.

22. Leigh Gilmore's work on the confessional and autobiography explores such intersections; see Leigh Gilmore, *The Limits of Autobiography: Trauma and Testimony* (Cornell, 2001).

23. Sidonie Smith and Julia Watson, *Reading Autobiography: A Guide For Interpreting Life Narratives* (Minneapolis, 2010), p. 17.

24. Smith and Watson, *Reading*, pp. 72–76.

25. Rebecca Steinitz, *Time, Space, and Gender in the Nineteenth-Century British Diary* (London, 2011), pp. 4–5.

26. A significant precedent for this approach is found in the memoirs or diaries of exceptional events, such as the Indian Rebellion of 1857; for example, in her diary of the Siege of Lucknow, Maria Germon writes: ‘The writer…wishes it to be understood that the Diary is in its original wording…with no attempts at embellishment’. Maria Germon, *A Lady's Diary During the Siege of Lucknow (*London, 1870), np.

27. Charles Grey, ‘Soldiering In Victorian Days: a Memoir and Sketches’, British Library, Mss Eur F391/2: 1941, 316.

28. David Gilmour, *The Ruling Caste: Imperial Lives in the Victorian Raj* (London, 2005), p. 55. Neil Bruniat Bonarjee, Mss Eur F180/72, BL, 2.

29. Cadambi Sheshachar Venkatachar, Mss Eur F180/85, BL, 3.

30. Venkatachar F180/85, 3, Unlike the pre-1914 format, Venkatachar thought that this system favoured the chances of Indian students, was responsible for his own success, and assisted the process of Indianisation of the government of India.

31. Victor George Matthews, Mss Eur F180/44, BL, 1.

32. Matthews, F180/44, 2. Matthews was motivated to join the ICS after the Wall Street Crash of 1929 and relates that when asked his reasons for applying he tells the truth rather than ‘the most welcome response’, which is ‘follow the family tradition’ or ‘I have a feeling for India and have an ambition to serve that part of the British Empire’. Apparently after explaining his desire for financial security there was ‘a long silence’ followed by the ‘rapid termination of my interview’.

33. Dewey, *Anglo-Indian Attitudes*, p. 5.

34. Dewey, *Anglo-Indian Attitudes*, p. 6.

35. Nasir Ahmad Faruqui, Mss Eur F180/27, BL, 6.

36. See Crane, *Inventing India*, p. 5; Bart Moore-Gilbert, *Writing India 1757-1990: The Literature of British India* (Manchester, 1996), p. 7.

37. See Humphrey Trevelyan, *The India We Left: Life as One of the Last Generation of British Officials in India* (London, 1972), pp. 112–13; Dewey, *Anglo-Indian Attitudes*, p. 211.

38. See Graham Huggan, *The Postcolonial Exotic: Marketing the Margins* (London, 2001); Elleke Boehmer, *Colonial & Postcolonial Literature* (Oxford, 1995).

39. For instance, Hugh Trevor Lambrick's account notes how his was one of the ‘service families’ of India, with relatives on his father's side in Bombay since 1805, and that his mother was a Trevor, described by Kipling as one of four most eminent dynasties in British India. Hugh Trevor Lambrick, Mss Eur F180/31, BL, 1.

40. Eric Atkinson Midgley, Mss Eur F180/78, BL, 5.

41. Ian Hay MacDonald, Mss EUR F180/20, BL, 1.

42. Sidney William Cecil Dunlop, Mss Eur F180/51, BL, 1.

43. Sir (Thomas Alexander) Fraser Noble, ‘Something in India. A memoir of service in the Frontier Province’, Mss Eur F226/18, BL, 8.

44. For instance, John Masters’ *Nightrunners of Bengal* (1951), J. G. Farrell's *The Siege of Krishnapur* (1973), and G. M. Fraser's *Flashman in the Great Game* (1975). For further detail on Rebellion fictions see Gautam Chakravarty, *The Indian Mutiny & the British Imagination* (Cambridge, 2005).

45. Midgley, F180/78, BL, 5.

46. Samuel Solomon, Mss Eur F180/24, BL, 13.

47. Although he is posted to Bihar, his third choice, Solomon nevertheless makes a pilgrimage to Lucknow at his first opportunity.

48. Harish Chandra Gupta, Mss Eur F180/10, BL, 5; Walter Geoffrey Lamarque, Mss Eur F180/54, BL, 2.

49. Forster's inclusion is in part paratextually motivated by the contemporaneous redefinition of modernism's place in the literary canon from the 1960s onwards; though modernism had been a fixture of English studies since the 1940s, Alex Murray argues that scholars and courses at the ‘new’ universities made a concerted effort to go beyond Eliot and Lawrence in the 1960s and 1970s. Similarly, Forster's death in 1970, and the posthumous publication of *Maurice* (1971), his first novel since *A Passage to India*, would have likely placed him at the forefront of these diarist's minds. See Alex Murray, ‘Changes in the Canon’, in Phillip Tew and Alex Murray (eds), *The Modernism Handbook* (London, 2009), p. 159.

50. Sir (Joseph) Herbert Thompson, (1898–1984), Indian Civil Service, Madras 1923–26, IPS 1926, Resident, Punjab States 1945-47 ‘Icarus went East’: memoirs of his career in the Royal Naval Air Service; ICS Madras; Dera… Mss Eur F226/29: 1916–1947, BL, 73.

51. Samuel Solomon, Mss Eur F180/24, BL, 13–14. Solomon's analysis is not a great advertisement for his legalistic training as Aziz is never convicted, and the charges against him are dismissed.

52. Thompson, F226/29, BL, 100.

53. Hugh Thompson Lane, Mss Eur F180/76, BL, 7.

54. Venkatachar, F180/85, BL, 49.

55. Richard Mercer Keene Slater, Mss Eur F180/69, BL, 6.

56. See Hywel Dix (ed.), *Autofiction in English* (London, 2018).

57. Not all contributors state their birth year, but those that are given range typically between c. 1900–1916. There are some earlier outliers (usually in the Political as they recruit from existing ICS or Military personnel e.g. Herbert Thompson, born 1898, or Geoffrey Ramsden, born 1893), but none of the respondents are born after 1916; this was the last intake of the ICS as a result of the combined postgraduate requirement of the service, the mandatory probationary year, and the hold on recruitment from 1939 as a result of the war. As mentioned above, those who had begun their probationary year by 1939 were still allowed to travel to India in 1940.

58. Petru Golban, *A History of the Bildungsroman: From Ancient Beginnings to Romanticism* (Newcastle-Upon-Tyne, 2017), p. 314.

59. Sarah Graham (ed.), *A History of the Bildungsroman* (Cambridge, 2019), p. 4.

60. Ericka A. Hoagland, ‘The Postcolonial Bildungsroman’, in Graham, *A History of the Bildungsroman*, p. 225.

61. Contemporary scholarship on the Raj Revival argues that its 1970s–1980s incarnation was just one iteration of a continual process of resurgence and recession in its popularity with the period around the United Kingdom's decision to leave the European Union once again prompting a return to the same questions of national identity through culture; see Sam Goodman, *The Retrospective Raj: Medicine, History and Literature After Empire* (Edinburgh, 2022).

